# A Review of High-Temperature Aerogels: Composition, Mechanisms, and Properties

**DOI:** 10.3390/gels10050286

**Published:** 2024-04-23

**Authors:** Conghui Wang, Letian Bai, Hongxin Xu, Shengjian Qin, Yanfang Li, Guanglei Zhang

**Affiliations:** 1 School of Materials Science and Engineering, Engineering Research Center of Matamaterials and Microdevices, Shijiazhuang Tiedao University, Shijiazhuang 050043, China; 1202308048@student.stdu.edu.cn (C.W.); 1202308001@student.stdu.edu.cn (L.B.); 1202208065@student.stdu.edu.cn (H.X.); qinsj@stdu.edu.cn (S.Q.); 2 National Engineering Research Center for Colloidal Materials, School of Chemistry and Chemical Engineering, Shandong University, Jinan 250100, China; 202384000220@sdu.edu.cn

**Keywords:** high-temperature aerogel, thermal insulation performance, mechanisms, application

## Abstract

High-temperature aerogels have garnered significant attention as promising insulation materials in various industries such as aerospace, automotive manufacturing, and beyond, owing to their remarkable thermal insulation properties coupled with low density. With advancements in manufacturing techniques, the thermal resilience of aerogels has considerable improvements. Notably, polyimide-based aerogels can endure temperatures up to 1000 °C, zirconia-based aerogels up to 1300 °C, silica-based aerogels up to 1500 °C, alumina-based aerogels up to 1800 °C, and carbon-based aerogels can withstand up to 2500 °C. This paper systematically discusses recent advancements in the thermal insulation performance of these five materials. It elaborates on the temperature resistance of aerogels and elucidates their thermal insulation mechanisms. Furthermore, it examines the impact of doping elements on the thermal conductivity of aerogels and consolidates various preparation methods aimed at producing aerogels capable of withstanding temperatures. In conclusion, by employing judicious composition design strategies, it is anticipated that the maximum tolerance temperature of aerogels can surpass 2500 °C, thus opening up new avenues for their application in extreme thermal environments.

## 1. Introduction

High-temperature insulation materials play a pivotal role in engineering systems and various industrial processes. Providing effective insulation at elevated temperatures is crucial for enhancing energy efficiency and achieving carbon neutrality objectives. Leveraging the porous and lightweight nature of aerogels, these materials offer exceptional thermal insulation properties. Compared to traditional insulation materials, aerogels exhibit remarkably low thermal conductivity (≤0.1 W/(m/K)). Moreover, aerogels demonstrate impressive resistance to corrosion and oxidation, showcasing outstanding chemical stability. Conventional silica aerogels typically remain stable within the range of 300 to 800 °C, which might fall short for demanding applications in military aerospace, energy-efficient construction, and electric vehicle batteries. Meeting the stringent demands of these diverse fields necessitates advancing aerogel technology. Aerogels meet the high-temperature engineering standards and also provide robust technical support for sustainable energy development.

Generally, the preparation process of aerogels includes sol-gel and drying processes. The sol-gel process entails the utilization of compounds rich in highly reactive chemical entities as precursors, which engage with solvents in reactions such as hydrolysis or alcoholysis. These reactions give rise to a stable and transparent sol matrix within the solution. As the sol matures, its constituent particles gradually undergo polymerization, evolving into a gel with a three-dimensional network structure. The crevices within this gel framework are permeated by solvent, resulting in the transition from fluid to a state lacking fluidity. Drying is a critical step in the preparation process, which aims to preserve the aerogel pore structure while removing the solvent. Freeze drying is a process that sublimates the solvent straight from a solid to a gas phase by freezing below its frozen point without creating a liquid phase. But, it is challenging to obtain an entire aerogel bulk due to the ice crystal growth effect. Supercritical drying utilizes the principle of the disappearance of the liquid–gas phase interfaces in supercritical fluid status, whereby the capillary force doesn’t influence the aerogel. It can better preserve the 3D skeleton and prepare the aerogel as a whole with a well-developed network structure, but this technique is costly and dangerous. Some rigorous definitions consider that solid aerogels obtained by supercritical drying are called aerogels, while those obtained by drying under non-supercritical conditions are xerogels.

**Figure 1:**
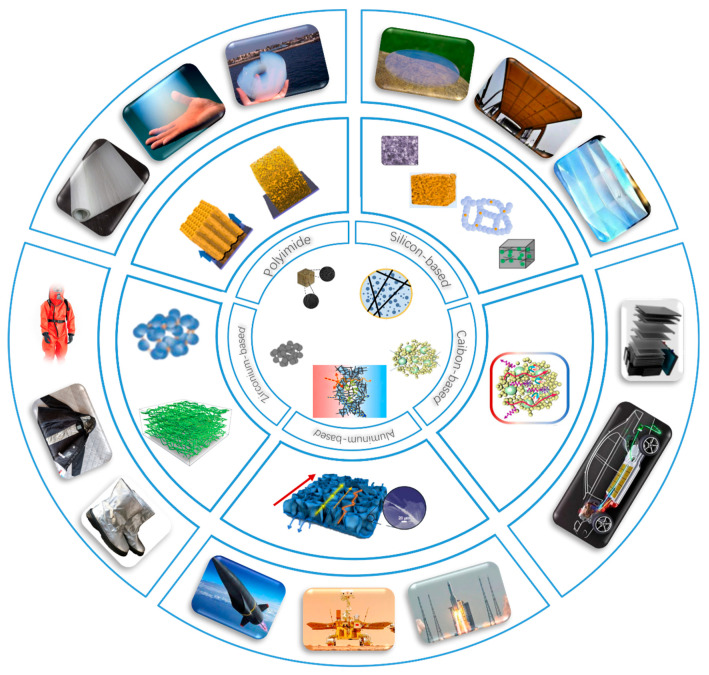
Classification and application of high-temperature aerogels.

High-temperature aerogels [1], classified based on their primary components, encompass silica (SiO_2_)-based, carbon-based, alumina (Al_2_O_3_)-based, polyimide-based, and zirconia (ZrO_2_)-based variants. Figure 1 provides a comprehensive overview of five categories of aerogels, detailing their respective doping elements, mechanisms of thermal insulation, and a wide range of practical applications. Each type of aerogel boasts distinct properties and application advantages. Silica-based aerogels, characterized by their low thermal conductivity (≤0.03 W/(m/K)), chemical stability, and thermal resilience, find applications in highly efficient thermal insulation contexts such as building insulation, pipe cladding, and thermal protection systems for space exploration [2]. For example, IBIH Advanced Material Co. Ltd. (Xuchang, Henan, China) provides the construction industry with a full range of energy-saving solutions based on aerogel technology to reduce energy consumption and improve the comfort of the living environment. Silica-based aerogels also break new ground in cryogenic liquid transportation [3]. Fiber-reinforced aerogel blankets (FRABs) provide superior insulation, reducing the boil-off rate of liquefied natural gas (LNG) significantly more efficiently than perlite. Traditional insulation materials like perlite have limitations that FRABs can potentially overcome. This improvement could lead to cost savings and more efficient use of space in LNG transport. Additionally, the use of FRABs could increase safety during installation and maintenance of the cryogenic tanks. Carbon-based aerogels, known for their high specific surface area, electrical conductivity, and thermal stability, are utilized in super capacitors, battery electrode materials, catalyst carriers, and adsorbents [4]. Fanruiyihui Composite Material Co., Ltd. (Zhengzhou, Henan, China) has innovatively deployed aerogel insulation mats in the new energy battery sector. Alumina-based aerogels exhibit thermal reflectivity and stable chemical properties, making them ideal for high-temperature thermal reflection insulation and catalyst carrier applications [5]. Polyimide aerogels offer outstanding mechanical properties, thermal stability, and low thermal conductivity, making them suitable for aerospace, electrical, and electronic applications. Zirconia-based aerogels boast high-temperature resistance and exceptional corrosion resistance, catering to specialized applications due to their unique physicochemical attributes. Shanghai Gaoguanda Material Technology Co., Ltd. (Shanghai, China) uses biomass and diverse industrial waste to produce aerogels [6], providing a more environmentally sustainable alternative while addressing the challenge of costly raw materials and manufacturing processes. Reutilizing waste materials can significantly reduce our environmental footprint and harness a tremendous repository of potential resources. This approach promotes a circular economy and a more environmentally friendly and sustainable future.

## 2. Classification and Characteristics of Thermal Insulation Materials

### 2.1. Silica-Based Aerogels

Silica aerogels, renowned for their exceptional lightness, high porosity, and outstanding thermal insulation properties, have emerged as a focal point in the field of material science in recent years. Currently, silica aerogels can withstand temperatures up to 1500 °C, and their thermal conductivity at room temperature is as low as 0.014 W/(m·K).

The preparation of silica-based aerogels usually involves the use of a silicon source as a precursor. At present, the common precursors of silica aerogels include tetraethoxysilane (TEOS), methyltrimethoxy-silane (MTMS), methyltriethoxysilane (MTES), and polymethyl-siloxane. However, only a few silica-based aerogels are high-temperature resistant, mostly aerogels obtained from organoalcoxysilanes. For example, MTMS or MTES are not resistant to high temperatures.

#### 2.1.1. Silica Aerogels

SiO_2_ aerogels are characterized by high porosity, low density, and low thermal conductivity, making them promising candidates for super-insulation materials. These aerogels exhibit the lowest thermal conductivities, rendering them suitable for temperatures up to 800 °C [7,8,9]. Jiang et al. [10] achieved an innovative breakthrough by synthesizing silica aerogels with a thermal conductivity of 0.02723 W/(m·K) through particle size variation using monodisperse silica sol. However, pure silica aerogels are limited to short-term usage at temperatures as high as 1100 °C. To further reduce thermal conductivity below 0.03 W/(m·K), Wang et al. [11] applied a surface modifier to produce dry, hydrophobic silica aerogels at ambient conditions. Despite their low thermal conductivity, silica aerogels encounter challenges such as brittleness, susceptibility to breakage, and alterations like shrinkage and deformation during processing [12]. To enhance properties such as strength, toughness, and high-temperature resistance, additional components are incorporated into the silica base to fabricate advanced high-temperature gels.

#### 2.1.2. Silica-Based Composite Aerogels

**Figure 2:**
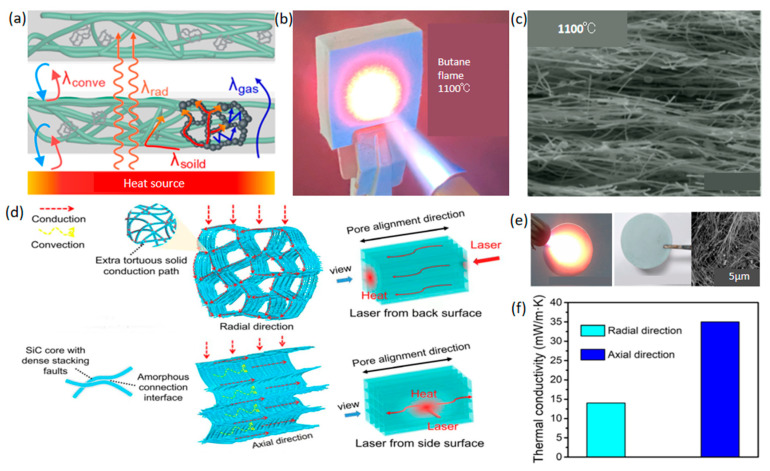
(**a**–**c**) Heat insulation mechanism and temperature resistance of ceramic nanofiber–particle composite aerogel. Adapted with permission from [21], copyright 2022, American Chemical Society: (**a**) Schematic illustration depicting factors contributing to the thermal conductivity of the ceramic nanofiber-particle composite aerogel. (**b**) Ceramic nanofiber–particle composite aerogel exposed to a butane blowtorch flame without any destruction. (**c**) Cross-sectional SEM images of the ceramic nanofiber–particle composite aerogel sintered at high temperatures up to 1100 °C. (**d**–**f**) Thermal insulation mechanism and temperature resistance of SiC/SiO_2_ nanowire aerogel. Adapted with permission from [22], copyright 2020, American Association for the Advancement of Science. (**d**) Schematic illustration demonstrating the mechanism for achieving thermal superinsulation. (**e**) The photograph and SEM image of the aerogel after being treated by the butane blowtorch for 30 min. (**f**) Thermal conductivities of the SiC/SiO_2_ nanowire aerogel in axial and radial directions.

Compared to pure silica aerogels, silica-based composite aerogels, enriched with various elements and compounds, exhibit significant enhancements in thermal stability and mechanical properties. Tang et al. [13] developed a SiO_2_ aerogel composite infused with a novel type of spherical, hollow infrared shading agent, demonstrating thermal conductivity ranging from 0.027 W/(m·K) to 0.050 W/(m·K) across temperatures from 300 K to 1400 K. Shen et al. [14] developed nano-SiO_2_ composites by wet impregnation of glass fiber felt with nano-SiO_2_ aqueous slurry and ambient pressure drying, showcasing a density of 0.24 g/cm^3^ and a thermal conductivity of 0.1334 W/(m·K) at 700 °C. Wang et al. [15] enhanced polymethyl-siloxane aerogels with minute amounts of polyimides, achieving a thermal conductivity of 0.016 W/(m·K). Additionally, Wang et al. [16] introduced a silica mineralized lignin nanocomposite aerogel, withstanding flames up to 1200 °C and exhibiting a thermal conductivity of 0.04 W/(m·K) under 33–94% relative humidity conditions. Gao et al. [17] utilized sol-gel and atmospheric pressure drying techniques to produce a composite aerogel with a specific surface area and pore volume of 179.5 m^2^/g and 1.295 cm^3^/g, respectively, at 1000 °C. Furthermore, Zhang et al. [18] created a ternary composite of Mullite fiber felt/emulsion/aerogel through impregnation, resulting in a low-density (0.195 g/cm^3^) material with a thermal conductivity of 0.042 W/(m·K). Additionally, Zhang et al. [19] formulated a novel Y_2_SiO_5_ trigenicity gel with thermal conductivities ranging from 0.029 to 0.05 W/(m·K). Su et al. [20] introduced a TiCN/SiBCN ceramic aerogel with a thermal conductivity of 0.08 W/(m·K), while Ding et al. [21] prepared ceramic nanofiber–particle composite aerogels, as illustrated in Figure 2a–c.

#### 2.1.3. Nanofiber Composite Aerogels

Nanofiber composite aerogels, compared to conventional aerogels, demonstrate superior mechanical properties and exceptional temperature resistance. Wang et al. [22] synthesized SiC/SiO_2_ nanowire aerogels featuring an anisotropic and layered microstructure assembled from microcrystals using directional freeze casting followed by heat treatment. SiC/SiO_2_ nanowire aerogels exhibit a thermal conductivity of approximately 0.014 W/(m·K) and maintain stability at 1200 °C, as shown in Figure 2d–f. The integration of pore structures and nanowire constituents creates numerous additional zigzag paths for solid conduction within the cross-section, effectively reducing radial heat transfer efficiency. This anisotropic thermal conductivity is evidenced by varying heat transfer behaviors across two principal directions, with the radial orientation demonstrating superior insulation capabilities. Additionally, Wang et al. [23] developed highly porous three-dimensional (3D) SiC/NWA materials with a density of 5 mg/cm^3^ and thermal conductivity of 0.026 W/(m·K). Similarly, Zhang et al. [24] fabricated a multifunctional SiC/SiO_2_ nanofiber aerogel with a 3D porous cross-linked structure, achieving a thermal conductivity of 0.027 W/(m·K). Zhou et al. [25] produced a composite aerogel of silanized cellulose nanofibers and hydroxylated boron nitride nano-sheets (Si/CNF/BNNS), displaying axial and radial thermal conductivities of 0.0621 W/(m·K) and 0.0339 W/(m·K), respectively. Zhang et al. [26] also developed glass fiber (GF) reinforced SiO_2_ aerogel composites, with a thermal conductivity of 0.0248 W/(m·K). Arnaud Rigacci and colleagues [27] developed a short cellulose fiber–silica composite aerogel, with the super critically dried (SC-dried) version exhibiting a thermal conductivity of 0.015 W/(m·K) and its ambiently dried equivalent at 0.017 W/(m·K). Yang et al. [28] introduced a nanofiber composite material with a thermal conductivity of 0.0251 W/(m·K). Furthermore, Yang et al. [29] devised a flexible SiO_2_ aerogel composite nanofiber film with a granular, strawberry-like structure through electrospinning technology, achieving a thermal conductivity of 0.0303 W/(m·K) and a surface area of 240 m^2^/g.

### 2.2. Carbon-Based Aerogels

Carbon-based aerogels, owing to their lightweight nature and high heat resistance, are ideal materials for the thermal protection of aerospace vehicles. At present, carbon aerogels are mainly made of carbon nanotubes, graphene, resorcinol, and other raw materials. The common precursors of carbon aerogels include carbon nanomaterials, organic polymer materials, and biomass materials. However, their brittleness and low mechanical strength currently hinder widespread application. To address these challenges, various tactics have been proposed, including altering doping elements, utilizing fiber composites, and more.

#### 2.2.1. Carbon-Based Aerogels with Doping Elements

**Figure 3:**
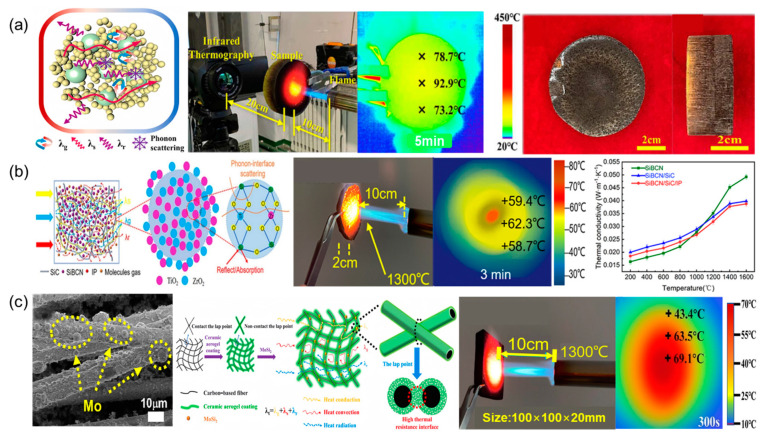
Fire Resistance and Thermal Insulation Mechanism Diagram of Carbon-Based Aerogel: (**a**) Schematic diagram illustrating the thermal insulation mechanism of SiCF/PR. Setup of the butane torch combustion test and thermographic pictures of the backside for a 3 cm thick sample after 5 min. Front and side views of the combustion test. Adapted with permission from [37], copyright 2022, Elsevier. (**b**) Schematic diagram illustrating the thermal insulation mechanism of SiBCN/SiC/IP. Adapted with permission from [40], copyright 2022, Elsevier. (**c**) Schematic illustration of the thermal insulation mechanism and optical photograph of the butane spray gun ablation test. Adapted with permission from [41], copyright 2023, Elsevier.

Typically, achieving high mechanical strength in carbon aerogels involves increasing bulk density or reinforcing them with fibers [30,31,32]. While increasing bulk density contributes to improved mechanical strength, it does not alter their inherent rigidity [33]. However, reinforcing carbon aerogels with fibers can enhance their elastic properties [34,35]. Both approaches increase the density of carbon aerogels, often accompanied by increased thermal conductivity, thus balancing the trade-off between thermal and mechanical constraints. Feng et al. [36] impregnated carbon aerogel composites (C/CA) into a silicon carbon oxide (SiCO) precursor sol multiple times, resulting in SiCO ceramic inner coating (C/CA/SiCO) anti-oxidation carbon aerogel composites with remarkable thermal stability. The weight loss for the as-prepared composites after heating at 1600 °C for 60 min in the air was only 7.6%, and the in-plane shrinkage was less than 2%. Zhang et al. [37] employed a robust multistage constitutive method to fabricate a novel needle carbon fiber-enhanced silicon carbon oxide-phenolic interpenetrating aerogel (SiCF/PR) nanocomposite, exhibiting outstanding thermal conductivity (0.068 W/(m·K)). Exposed to a blowtorch flame, the center temperature on the front side of the sample rose to about 1000 °C quickly but the backside temperature only increased 92.9 °C after 5 min (Figure 3a). During the whole heating process, the samples exhibit excellent non-combustibility and dimensional stability without any obvious size and shape changes. Guo et al. [38] reported a high-pressure-assisted polymerization method combined with ambient pressure drying to produce carbon/carbon (C/C) composites with exceptional resistance to oxyacetylene flame exposure. Feng et al. [39] proposed a carbon layer encapsulation (CLE) strategy to tailor multifunctional aerogels, resulting in a unique Al_2_O_3_-C core–shell binary synergistic structure with impressive thermal conductivity.

#### 2.2.2. Carbon Fiber Aerogels

Carbon-based fibers possess high strength, low density, and excellent thermal stability [42,43,44]. However, at high temperatures, agglomeration of particles and collapse of pores may compromise the oxidation resistance of ceramic aerogels. Li et al. [40] introduced disilicate nanoparticles into nanofibers through precursor infiltration pyrolysis (IP) and electrospinning technology, resulting in a finger coral-like SiCBN/SiC/IP fiber-reinforced aerogel with improved mechanical properties. Through the butane spray gun insulation test, they discovered the temperature on the back of SiBCN/SiC/IP gradually increased and remained at a relatively stable temperature (~75 °C) after 10 min and the thermal conductivity of it increased slowly alongside the test temperature (Figure 3b); the thermal conductivity at 1600 °C is only 0.036 W/(m·K). Zhang et al. [45] constructed a ceramic aerogel coating on carbon-based fiber surfaces using impregnation and polymer-derived ceramics, achieving a carbon-based fiber aerogel with a high thermal resistance interface (CE/SiCF/Mo) and exceptional high-temperature insulation performance. As a result, the thermal conductivity of the composite is only 0.025 W/(m·K). Remarkably, the aerogel exposed to a butane flame at 1300 °C for 5 min displayed a very low back-side temperature of 69.1 °C and exhibited excellent high-temperature insulation performance. Zhang et al. [45] and [46] developed carbon aerogel composites with short carbon fibers and a unique lightweight heat protection and insulation integrated composite (QCF/SPA), respectively, employing innovative fabrication strategies. The composite aerogel is capable of retaining its excellent pore structure even at temperatures as high as 2500 °C, with a remarkably low thermal conductivity of only 0.05 W/(m·K).

#### 2.2.3. Graphene Aerogels

In recent years, various innovative carbon nanostructured materials have been synthesized and extensively studied to meet the demands of novel applications [47,48]. Three-dimensional graphene aerogels (GA) exhibit flexibility, strong mechanical strength, lightweight, high porosity, and excellent durability. While single-layer graphene aerogels have positive thermal conductivity, the nano-porous structure, low density, and perfect opacity of graphene aerogels suppress gaseous, solid, and radiant thermal conductivity. Additionally, defects in graphene and the relatively small sizes of graphene sheets further reduce thermal conductivity. Xie et al. [49] prepared graphene aerogels with ultra-low thermal conductivities (0.0047–0.005.9 W/(m·K)), demonstrating superior thermal insulation properties. Zhang et al. [50] prepared graphene aerogels with improved mechanical properties and thermal conductivity via hydrothermal reduction and supercritical ethanol drying, making them promising candidates for various applications spanning from batteries to pressure sensors, electrodes, lightweight conductors, and insulation materials.

### 2.3. Alumina-Based Aerogels

Alumina-based aerogels are primarily composed of aluminum sec-butoxide, aluminum acetylacetonate, water, and ethanol as raw materials, and aerogels are obtained through supercritical drying. Despite exhibiting good thermal stability, alumina-based aerogels undergo a series of phase transitions at elevated temperatures, leading to volume shrinkage and compromising their nano-porous structure. To address this issue, current efforts focus on improvements in microstructure, doping elements, and the addition of light-blocking agents. In recent years, significant progress has been made in the research of alumina-based aerogels. These aerogels exhibit low thermal conductivity and can maintain their nano-pore structure at temperatures up to 1000 °C, rendering them suitable for applications in aerospace and industrial kilns.

#### 2.3.1. Alumina Aerogels

Alumina aerogel, characterized by its nano-porous structure with high specific surface area, low density, and low thermal conductivity, has garnered considerable attention. Feng et al. [51] obtained alumina aerogels with a specific surface area of 95 m^2^/g through hydrothermal treatment and supercritical drying, while Shen et al. [52] synthesized alumina aerogels with a specific surface area of 154 m^2^/g using acetone–aniline hydrogels. Various methods for preparing and controlling the performance of alumina aerogels have been introduced. For instance, Feng et al. [53] utilized direct hydrothermal treatment and supercritical drying to adjust the crystallinity of boehmite colloid, resulting in heat-resistant and well-crystallized alumina aerogels. Shen et al. [54] prepared high surface area alumina aerogel monomers without complexing agents, employing the sol-gel method combined with ethanol supercritical drying technology, yielding alumina aerogels with a specific surface area of 690 m^2^/g. Subsequent heat treatment at 800 °C transformed them into γ-Al_2_O_3_, with no significant loss of surface area.

#### 2.3.2. Alumina–Silica Aerogels

**Figure 4:**
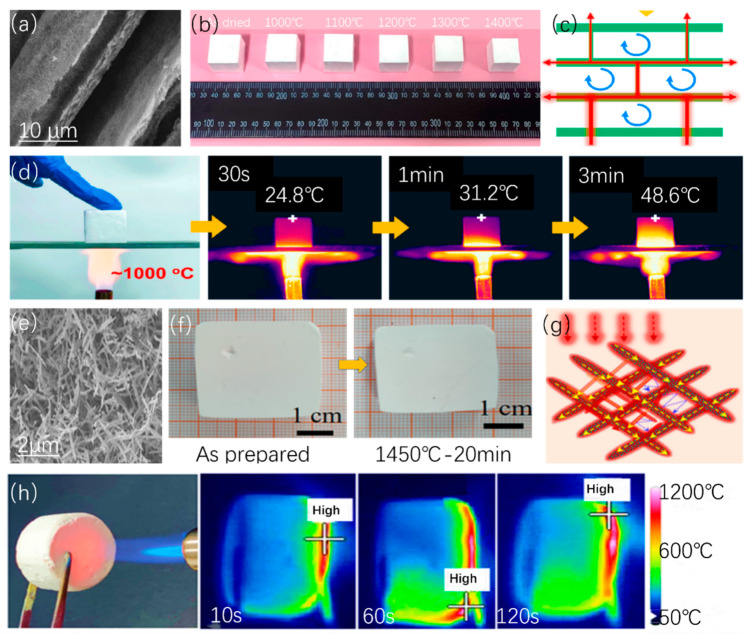
Fire resistance of sheet and rod-shaped aluminum–silica aerogels. (**a**) Scanning electron microscope image of sheet aerogels. (**b**) Thermal shrinkage of the sample at different temperatures. (**c**) Thermal insulation mechanism. (**d**) Thermal imaging of the combustion experimental device and sample. Adapted with permission from [55], copyright 2011, Elsevier. (**e**) Scanning electron microscope image of rod-shaped aerogel. (**f**) Thermal shrinkage of the sample after heating for 20 min. (**g**) The thermal insulation mechanism of rod-shaped CE/SiCF/Mo aerogel. (**h**) Thermal imaging of the combustion experimental device and samples. Adapted with permission from [57], copyright 2023, American Chemical Society.

Alumina–silica (Al_2_O_3_/SiO_2_) aerogels have advanced in structural design. Feng et al. [55] developed double-template nano porous ceramic aerogels, resistant to temperatures up to 1000 °C. Figure 4a–d shows the sample’s cold side temperature was 45.6 °C after heating at 1000 °C for 180 s. Chen et al. [56] utilized silica sol as a high-temperature binder to produce alumina aerogels with high-temperature resistance and low linear shrinkage. Zhang et al. [57] fabricated a high-temperature-resistant alumina nanorod aerogel (Figure 4e–h) with a self-supporting structure and strong Si-O-Al bond, exhibiting exceptional performance even at 1200 °C. After heating at 1200 °C for 120 s, the sample’s cold side temperature in Figure 4h was about 50 °C, showing its high-temperature resistance. Guo et al. [58] designed ceramic nanofiber aerogels with a layered multi-arch structure for use in high-temperature environments. Innovative preparation methods for alumina–silica aerogels have been proposed. Feng et al. [59,60] introduced a new strategy of sol-impregnation-gel and supercritical drying, along with a supercritical drying method after hydrothermal treatment. Shen et al. [61] synthesized TiO_2_/fiber/alumina-based aerogel ternary composites by sol-gel method, impregnation method, and thermal insulation drying method. Moreover, traditional sol-gel methods [62] have been employed to produce aerogels with low thermal conductivity and high strength after calcination. However, alumina–silica aerogels [45] have exhibited superior performance at high temperatures, with high-temperature resistance achievable by adjusting the molar ratio of the alumina–silica composite aerogel [63].

Furthermore, alumina and silicon oxide can form mullite, with a melting point up to 1800 °C, commonly used in high-temperature furnace refractory materials and linings. Hou et al. [64] synthesized a special crystal phase of mullite, while Shen et al. [65] developed chelator-free monolithic mullite fiber-reinforced alumina aerogels, exhibiting remarkable mechanical properties after treatment at 1300 °C. Al_2_O_3_/SiO_2_ aerogels prepared with water glass and aluminum chloride as raw materials [66] produced mullite crystals during heat treatment, enhancing high thermal stability. Mullite fiber-filled aluminum hydroxide sol yielded mullite fiber/aluminum oxide composite aerogels [67], and aluminum silicate nanofibers integrated into Al_2_O_3_/SiO_2_ aerogels [68] further improved performance.

#### 2.3.3. Other Alumina-Based Aerogels

By doping different elements, the structure and properties of aluminum-based aerogels can be adjusted to achieve multifunctionality. Feng et al. [69] prepared a multi-scale fiber-reinforced Al_2_O_3_/C core–shell aerogel composite with a thermal conductivity of 0.055 W/(m·K) at 1200 °C. Wang et al. [70] developed alumina–silica aerogels doped with 1.0 wt% carbon nanotubes, exhibiting a thermal conductivity of 0.178 W/(m·K) at 1000 °C. Shen et al.’s research team [71] successfully synthesized alumina aerogels using the cross-linking method of organic/inorganic double precursors, achieving a surface area of 105 m^2^/g after heating at 1300 °C. Ding et al. [72] designed multiphase ceramic nanofibers, demonstrating multi-dimensional flexibility under high-temperature environments. Lin et al. [73] prepared AlCl_3_-chitosan composite aerogels by a solution freeze-drying technology, exhibiting a thermal conductivity of about 0.039 W/(m·K) after treatment at 1000 °C. Zhao et al. [74] produced sepiolite/alumina aerogel composites by the sol-gel method and supercritical fluid drying method, with a linear shrinkage of 4.6% after heat treatment. Ding et al. [75] combined ZrO_2_/Al_2_O_3_ fiber with Al (H_2_PO_4_)_3_ to form aerogels with a thermal conductivity of 0.0322 W/(m·K). Al_2_O_3_/Y_2_O_3_/SiO_2_ trigenics [76] exhibited a high specific surface area and a thermal conductivity as low as 0.079 W/(m·K) at 1000 °C. The new monolithic mullite aerogel [77] prepared by resorcinol/formaldehyde/Al_2_O_3_/SiO_2_ aerogel displayed excellent properties. Thermal insulation materials play a vital role in the thermal protection of aerospace spacecraft. For example, aluminum–carbon aerogel materials with a carbonization temperature of 800 °C [78] exhibited a weight loss of about 10% in the rmogravimetric tests. The Al_2_O_3_/SiO_2_ aerogel composite [79], prepared by sol-gel and supercritical drying technology and doped with varying contents of carbon nanotubes, demonstrated improved comprehensive performance.

### 2.4. Zirconia-Based Aerogels

Zirconia aerogels, with their high stability and superior temperature resistance, are at the forefront of research into the next generation of high-performance materials. Currently, zirconia aerogels boast a maximum temperature resistance of 1300 °C and a thermal conductivity of 0.104 W/(m·K).

**Figure 5:**
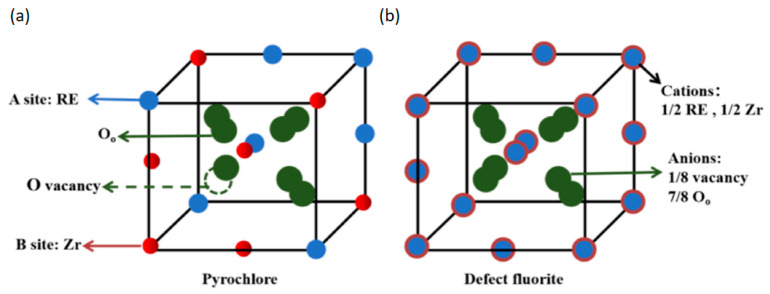
Pyrochlore (**a**) and defect fluorite (**b**) structure diagrams. Adapted with permission from [80], copyright 2022, Elsevier.

Zirconia aerogel, hailed as a high-tech material for its unique properties and extensive applications, has attracted considerable attention from both scientific researchers and industrial sectors in recent years. Li et al. [80] developed ceramic nanofiber aerogels incorporating amorphous carbon within yttrium-stabilized zircon nanofibers, achieving a notable thermal conductivity of 0.095 W/(m·K) at 1000 degrees Celsius. Meanwhile, Xu et al. [80] introduced a groundbreaking high-entropy rare earth zirconate (Sm_0.2_EuTbDyLu)_2_ZrO_7_ ceramic aerogel, exhibiting a remarkable thermal conductivity of 0.031 W/(m·K) post heat treatment at 900 °C. As illustrated in Figure 5, zirconium resides within the crystal lattice alongside five rare earth elements (samarium, europium, terbium, dysprosium, and lutetium), forming a defect fluorite structure characterized by disordered cation arrangement and oxygen vacancies. This structural intricacy elevates lattice complexity and compositional disorder, resulting in varied mass, charge imbalance, and chemical bond vibrations, ultimately leading to reduced thermal conductivity.

Furthermore, Liu et al. [81] synthesized a ZrC/C aerogel demonstrating thermal conductivities ranging between 0.0896–0.1064 W/(m·K). Additionally, Zhang et al. [82] successfully produced zirconia aerogels capable of preserving their nanoporous structure even at 1000 °C, characterized by a density of 0.074–0.136 g/cm^3^ and a specific surface area of 650 m^2^/g.

Through the integration of silicon elements and innovative architectural designs, material temperature resistance has been further bolstered. Li et al. [9] engineered a three-dimensional (3D) zircon fiber matrix, developing a multi-scale sub-crystalline zircon nanofiber aerogel with a sawtooth structure, achieving a commendable thermal conductivity of 0.104 W/(m·K) at operational temperatures of 1000 °C and designed for use up to 1300 °C. Similarly, Ding et al. [83] constructed flexible ZrO_2_/SiO_2_ nanofibers into a fluffy, layered arch honeycomb structure, resulting in an aerogel thermal conductivity of 0.0268 W/(m·K). Moreover, the dimethylldiethoxysilane-modified ZrO_2_/SiO_2_ aerogel prepared by Wu et al. [84] further reduced thermal conductivity to 0.02332 W/(m·K).

Additionally, doping zirconium aerogels with aluminum has yielded promising outcomes. Ding et al. [73] introduced a layered multi-arch ceramic nanofiber aerogel, where the ZrO_2_/Al_2_O_3_ nanofiber aerogel demonstrated remarkable high-temperature resistance up to 1300 °C and a thermal conductivity of 0.0322 W/(m·K) [85]. Meanwhile, Wu et al. [86] employed foldable all-ceramic air filtration materials for particle removal from high-temperature exhaust gases, with Al_2_O_3_-stabilized ZrO_2_ (ASZ) submicron fiber air filter paper exhibiting flexibility and thermal stability at 1100 °C.

The standout characteristic of zircon-based exothermic gel materials lies in their primary component, zirconium, renowned for its exceptional thermal properties, high-temperature resistance, and chemical stability. These attributes open up a wide array of applications for zirconium-based exothermal gels across various fields.

### 2.5. Polyimide Aerogels

Polyimide aerogels represent three-dimensional porous materials composed of cross-linked polymer molecular chains that amalgamate the excellent properties of polyimides and aerogels. These aerogels exhibit ultra-low density and low thermal conductivity. Due to their unique molecular structure, polyimides pose challenges in dissolving in many organic solvents for aerogel fabrication. A viable approach involves introducing hydrophilic units through structural design to enable polyimide dissolution or preparing polyimide aerogels via imidization starting from polyamic acid [87,88]. However, the former method proves expensive and difficult to scale up for large-scale production.

Supercritical CO_2_ drying stands as the primary method for aerogel fabrication [89,90,91,92]. Nonetheless, the high operational costs of supercritical CO_2_ and the uniform pore structure limitations restrict their widespread application [93,94,95]. In contrast, vacuum freeze-drying emerges as a promising alternative method due to its cost-effectiveness and environmental friendliness. Zou et al. [96] synthesized Polyimide/carboxyl functionalized multi-walled carbon nanotube (PI/MWCNTs/COOH) composite aerogels featuring an aligned slit-like channel structure through unidirectional freeze-drying. The incorporation of carbon nanotubes enhances the rigidity of polyimide molecular chains, reducing its liquidity and increasing its char yield to 56.1 wt% at 800 °C. Zhao et al. [97] reported a series of all-aromatic anisotropic polyimide aerogels (p-phenylenediamine (PDA)) fabricated by random freeze-drying, exhibiting a thermal conductivity of 0.0559 W/(m·K). This study also confirmed that unidirectional freeze-drying to prepare aerogels resulted in significantly lower thermal conductivity in the radial direction compared to the axial direction, whereas random freeze-drying yielded almost identical thermal conductivity in both directions.

## 3. Summary and Prospects

In general, single-component metal oxide aerogels tend to exhibit poor heat resistance. Currently, silica-based aerogels exhibit exceptional thermal resistance up to 1500 °C, boasting a remarkably low thermal conductivity of just 0.014 W/(m·K) at 1200 °C. Notably, Al_2_O_3_-SiO_2_ composite aerogels exhibiting a mullite structure have demonstrated enhanced thermal stability, withstanding temperatures of up to 1400 °C. In contrast, carbon-based aerogels demonstrate robust integrity, retaining their shape after being subjected to an oxyacetylene flame at a staggering 2500 °C for 30 s, while achieving a thermal conductivity of 0.05 W/(m·K) at 1200 °C. Additionally, aluminum-based aerogels showcase superior temperature resistance up to 1800 °C, with a thermal conductivity of 0.03274 W/(m·K), illustrating their high-performance characteristics in extreme thermal applications. Zirconia-based aerogels can endure up to 1300 °C with a thermal conductivity of 0.104 W/(m·K). Polyimide aerogels, with a maximum temperature tolerance of 1000 °C, have a thermal conductivity of 0.344 W/(m·K) at room temperature. These materials exemplify the significant advancements in aerogel technology, offering diverse applications due to their varied thermal resistances and conductivities. For instance, zirconia aerogels typically undergo significant shrinkage when subjected to annealing temperatures above 600 °C. In efforts to broaden the scope of aerogel applications, particularly in high-temperature (>650 °C) settings, significant attention has turned towards multi-component composite aerogels owing to their remarkable thermal stability. The thermal resistance of metal oxide aerogels, when composited with silica nanoparticles, has seen significant improvement. Incorporating fibers with exceptional thermal stability, such as mullite or sepiolite, serves as a viable strategy for reinforcing metal oxide aerogels, resulting in higher strength and reduced shrinkage. These composite materials hold promising applications in high-temperature thermal insulation.
